# Simple ammonium salts acting on sigma-1 receptors yield potential treatments for cancer and depression

**DOI:** 10.1038/s41598-020-65849-6

**Published:** 2020-06-08

**Authors:** James M. Brimson, Kiran K. Akula, Haider Abbas, David R. Ferry, Shrinivas K. Kulkarni, Steven T. Russell, Michael J. Tisdale, Tewin Tencomnao, Stephen T. Safrany

**Affiliations:** 1 Age-Related Inflammation and Degeneration Research Unit, Department of Clinical Chemistry, Faculty of Allied Health Sciences, Chulalongkorn University, Bangkok, 10330 Thailand; 2 Department of Pharmacy and Pharmacology, University of Bath, Claverton Down, Bath, BA2 7AY UK; 3 University Institute of Pharmaceutical Sciences, Panjab University, Chandigarh, 160014 India; 4 Present Address: Ultragenyx Pharmaceuticals Inc, Novato, California, CA94949 USA; 5 Department of Pharmacy, University of Wolverhampton, Wulfruna Street, Wolverhampton, WV1 1LY UK; 6 New Cross Hospital, Wolverhampton, WV10 0QP UK; 7 Present Address: University Hospital Birmingham NHS Foundation Trust, Mindelsohn Way, Birmingham, B15 2TH UK; 8 Eli Lilly, 893 S Delaware Street, Indianapolis, IN 46225 USA; 9 School of Health and Life Sciences, Aston University, Birmingham, B4 7ET UK; 10 RCSI-Bahrain, PO Box 15503 Adliya, Kingdom of Bahrain

**Keywords:** Chemotherapy, Receptor pharmacology

## Abstract

Sigma-1 and sigma-2 receptors are emerging therapeutic targets. We have identified that simple ammonium salts bind to these receptors and are effective *in vivo*. Radioligand binding assays were used to obtain structure-activity relationships of these salts. MTS assays were performed to determine their effect on growth in MCF7 and MDA-MB-486 cells. Anticancer properties were tested in NMRI mice transplanted with a fragment of mouse adenocarcinoma (MAC13). Antidepressant activity was tested using the forced-swim test and tail suspension tests. Dipentylammonium (K_i_ 43 nM), tripentylammonium (K_i_ 15 nM) and trihexylammonium (K_i_ 9 nM) showed high affinity for the sigma-1 receptor. Dioctanoylammonium had the highest affinity (K_50_ 0.05 nM); this also showed the highest affinity for sigma-2 receptors (K_i_ 13 nM). Dipentylammonium was found to have antidepressant activity *in vivo*. Branched-chain ammonium salts showed lower affinity. Bis(2-ethylhexyl)ammonium (K_50_ 29 µM), triisopentylammonium (K_50_ 196 µM) and dioctanoylammonium showed a low Hill slope, and fitted a 2-site binding model for the sigma-1 receptor. We propose this two-site binding can be used to biochemically define a sigma-1 receptor antagonist. Bis(2-ethylhexyl)ammonium and triisopentylammonium were able to inhibit the growth of tumours *in vivo*. Cheap, simple ammonium salts act as sigma-1 receptor agonists and antagonists *in vivo* and require further investigation.

## Introduction

The sigma opioid receptor was proposed as a novel fourth opioid receptor in 1976 to account for the behavioural effects of N-allylnormetazocine (SKF-10,047) which could not be accounted for by the μ (morphine) receptor or κ (ketocyclazocine) receptor^1^. Rather than causing analgesia, as with morphine, benzomorphans such as SKF-10,047 and pentazocine cause psychotomimesis. Martin proposed that SKF-10,047 was acting as a sigma opioid receptor agonist, in order to cause the psychological and behavioural effects which were observed^1^. It is now recognized that there are two sigma receptors: The sigma-1 receptor consists of a 223 amino acid long protein, which shares no homology with any other known mammalian protein^2^. X-ray crystallography analysis of the sigma-1 receptor has revealed a trimeric structure, with each receptor in the trimer having a single transmembrane domain anchoring it to the cytosolic side of the endoplasmic reticulum^3^. N,N-dimethyltryptamine may be the endogenous sigma-1 receptor ligand despite having low affinity for the sigma-1 receptor^4^. TMEM97 has now been proposed to be the unrelated “sigma-2 binding site”^5^.

The sigma-1 receptor has been linked with many diverse disease states and conditions. Sigma-1 receptor agonists appear to remedy symptoms of many mental conditions including depression, Alzheimer’s and drug addiction. Conversely, antagonists at the sigma-1 receptor can be used in the diagnosis and treatment of many cancers; in addition they also have analgesic properties^6^.

The efficacy of sigma-1 agonists in the treatment of depression has been recognized retrospectively, as many widely used antidepressants have now been shown to also act at the sigma-1 receptor^7^. Fluoxetine has been found to bind the sigma-1 receptor with reasonable affinity (214 nM)^8^, although affinity for the 5-HT reuptake transporter was much higher (0.81 nM)^8^.

Whilst the sigma-1 receptor has been mostly studied for its functions within the nervous system, it is also found to be very highly expressed in a wide range of human tumours that originate from both neuronal and non-neuronal tissues^9^. This has facilitated the clinical study of cancer through the application of sigma ligands in tumour imaging^10^, and the identification that sigma-1 receptor antagonists are able to prevent tumour growth, through calcium signalling, phospholipase C (PLC) activation, ER stress, and caspase activation^11,12^.

Development of new agents is costly and time-consuming. Leading chemists claim that each new chemical entity in drug discovery requires multiple synthetic steps (seven or more) for their production^13^. There have been a number of previous studies looking for a structural relationship between sigma-1 receptor ligands and their affinity for the sigma-1 receptor^14,15^. The importance of the nitrogen atom has been shown for the phenylalkylpiperidines and phenylalkylpiperazines^14,16^. However, evidence exists that more simple molecules can show high affinity for the sigma-1 receptor^17,18^.

We now determine that simple ammonium salts show clear structure-activity relationships for the sigma-1 receptor. Our simple ammonium salts differ from commonly used sigma-1 receptor ligands, which often contain more than one nitrogen and have complex aromatic ring structures; the simple ammonium salts contain only one nitrogen, and have simple carbon chains. These compounds were found to have high affinity for the unrelated sigma-2 receptor, TMEM97^5^. All but one of the straight-chained ammonium salts described show agonist-like profiles during *in vitro* studies. One of these, dipentylammonium, is shown to have antidepressant activity *in vivo* despite showing little activity towards transporters of 5-hydroxytryptamine (serotonin), dopamine or noradrenaline. Furthermore, the branched-chain ammonium salts showed antagonist-like properties during *in vitro* studies and the two tested were able to prevent tumour growth *in vivo*.

## Results

### Binding to the sigma-1 and sigma-2 receptors

Previous data have shown that MDA-MB-468 cells contain high concentrations of sigma-1 receptors (pK_d_ ± SEM of [^3^H] (+) pentazocine 7.8 ± 0.6, K_d_ 17 nM, B_max_ 2300 ± 200 fmol.mg^−1^)^19^. In contrast, no [^3^H] (+) pentazocine binding was observed in MCF7 cells, identifying these cells to be a good model to study sigma-2 receptor binding. This is in agreement with previous studies^9^. The binding of [^3^H] DTG to MCF7 cells showed a pK_d_ ± SEM of [^3^H] DTG 7.92 ± 0.04, K_d_ = 12 nM, B_max_ 1700 ± 400 fmol.mg^−1^. We have recently shown that [^3^H] DTG binding is reduced by (+) pentazocine with low affinity, further confirming the absence of sigma-1 receptors from MCF7 cells^20^.

This systematic study compared affinities of 34 ammonium salts for the sigma-1 and sigma-2 receptors. Those of interest were further assessed for cross-reactivity with other systems and *in vivo* biological activity.

Primary ammonium salts showed increasing affinity associated with increasing molecular weight. The highest affinity primary ammonium salt we tested was decylammonium with a K_i_ of 310 nM (Table 1, Supplementary Table ST1, Supplementary Fig. SF1). Affinity for the sigma-2 receptor showed a similar graded but less dramatic improvement. Decylammonium was found to have the highest affinity with a K_i_ of 3.9 µM (Table 1, Supplementary Table ST1, Supplementary Fig. SF1).

**Table 1:** Affinities of key ammonium salts tested.

Ammonium salt	σ-1 R affinity pK_i_ ± SEM (n)	σ-1 R affinity (µM)	σ-2 R affinity pK_i_ ± SEM (n)	σ-2 R affinity (µM)	σ-1 R K_i_/σ-2 R K_i_
Octyl	6.1 ± 0.3 (4)	0.80	5.2 ± 0.2 (3)	6.2	0.13
Decyl	6.5 ± 0.4 (4)	0.32	5.41 ± 0.07 (3)	3.9	0.081
Dipropyl	5.6 ± 0.2 (6)	2.6	4.10 ± 0.07 (3)	79	32
Dibutyl	6.9 ± 0.4 (6)	0.14	5.64 ± 0.07 (3)	2.3	18
Dipentyl^17^	7.4 ± 0.5 (6)	0.043	6.9 ± 0.2* (3)	0.12	3.2
Dihexyl	6.8 ± 0.6 (6)	0.14	7.58 ± 0.06* (3)	0.026	0.17
Dioctyl	10.3 ± 0.2 (6)	0.000050	7.9 ± 0.5 (3)	0.014	0.0040
Tributyl	6.9 ± 0.1 (6)	0.13	5.5 ± 0.2* (3)	3.0	0.040
Tripentyl	7.8 ± 0.2 (6)	0.015	6.1 ± 0.1 (3)	0.78	0.020
Trihexyl	8.1 ± 0.4 (6)	0.009	6.63 ± 0.03 (3)	0.23	0.034
Bis(2-ethylhexyl)	4.5 ± 0.3* (5)	29	4.0 ± 0.2* (3)	91	0.29
Triisobutyl	5.1 ± 0.6 (6)	7.9	5.46 ± 0.06 (3)	3.5	2.3
Triisopentyl	4.1 ± 0.4* (6)	79	6.40 ± 0.09* (3)	0.40	200
Tetrapropyl	2.5 ± 0.1 (3)	3,200	3.5 ± 0.2* (3)	310	10
Tetrabutyl	2.8 ± 0.1 (3)	1,600	4.22 ± 0.08* (3)	60	25

Affinities for the sigma-1 and sigma-2 receptors (σ-1 R and σ-2 R, respectively) are shown as pK_i_ and K_i_ (µM) values from competition binding assays. Hill slopes were determined as unity for all salts tested, except those labelled *. σ-1 R K_i_/σ-2 R K_i_ shows the ratio between K_i_ values. Sigma-1 receptor affinity has been previously published^17^. These data are reproduced from the PhD theses by J.M. Brimson^44^ and H. Abbas^45^.

Secondary ammonium salts bound with much higher affinity to the sigma-1 receptor. Affinity for the sigma receptors increased with chain length, with dioctylammonium showing the highest affinity (K_50_ 0.05 nM and 13 nM, for sigma-1 and sigma-2 receptors respectively) (Table 1, Supplementary Table ST1, Supplementary Fig. SF2). Of the compounds tested, dioctylammonium showed the greatest selectivity (245-fold) towards the sigma-1 receptor over the sigma-2 receptor. The binding of dioctylammonium to sigma-1 receptors showed a particularly low Hill slope (0.43 ± 0.02, mean ± SEM, n = 4). A two-site binding model fits the data better (Supplementary Fig. SF3), giving pK_i_ high 11.9 ± 0.3 (50 ± 7% of total binding) and pKi low 9.4 ± 0.2.

Tertiary ammonium salts also showed a graded increase in affinity. Tripentylammonium and trihexylammonium were found to have the highest affinity with K_i_ values for the sigma-1 receptor of 15 nM and 9 nM, respectively. The affinity for sigma-2 receptors showed a very similar pattern; trihexylammonium was found to have highest affinity with K_i_ of 230 nM. Increasing the chain length above six carbons was detrimental to binding to both sigma-1 and sigma-2 receptors (Table 1, Supplementary Table ST1, Supplementary Fig. SF4).

All quaternary ammonium salts tested had low affinity for the sigma-1 receptor. Tetrabutylammonium bound with highest affinity with a K_i_ of 1.6 mM, and there was little increase in affinity as the carbon chain length increased, therefore no larger quaternary ammonium salts were tested for sigma-1 receptor affinity. Similarly, quaternary ammonium salts showed low affinity for the sigma-2 receptor (Table 1, Supplementary Table ST1, Supplementary Fig. SF5).

In addition to these straight-chained ammonium salts, 10 branched-chain salts were tested. These tended to have lower affinity for the sigma-1 receptor than comparable straight-chained analogues (Table 1, Supplementary Table ST1, Supplementary Fig. SF6). Their affinity for the sigma-2 receptor was not as greatly reduced. Triisopentylammonium showed the greatest selectivity towards the sigma-2 receptor, with a 200-fold higher affinity for sigma-2 receptors over sigma-1 receptors. The binding of most straight-chained ligands to the sigma-1 receptors showed a simple displacement with a Hill slope of unity. In contrast, the branched-chain ammonium salts appeared to have shallower Hill slopes. A one-sample t test (GraphPad Prism) showed three had a Hill slope significantly less than unity at the sigma-1 receptor: bis(2-ethylhexyl)ammonium (0.24 ± 0.14, *P* = 0.001, n = 5), di-sec-butylammonium (0.41 ± 0.12, *P* = 0.040, n = 3) and triisopentylammonium (0.42 ± 0.12, *P* = 0.008, n = 6). Dicyclohexylammonium showed a low, but not statistically significant Hill slope (0.4 ± 0.2, *P* = 0.052, n = 5). The binding of bis(2-ethylhexyl)ammonium and triisopentylammonium was found to fit a 2-site model in preference to a one-site fit. Bis(2-ethylhexyl)ammonium was found to have pK_i_ high 6.4 ± 0.5 (18 ± 6% of total binding) and pK_i_ low 4.4 ± 0.3. Similarly, triisopentylammonium was found to have pK_i_ high 5.6 ± 0.4 (70 ± 13% of total binding) and pKi low 1.8 ± 0.2. (Supplementary Fig. SF3). Di-sec-butylammonium was found to best fit a one-site model.

The Hill slopes of binding to the sigma-2 receptor showed less of a pattern: eleven of the 34 tested showed a Hill slope significantly below unity (Supplementary Table ST1). All agents were found to best fit a one-site binding model at the sigma-2 receptor. The high affinity of some of these simple ligands led us to further examine their profile is a series of tests.

### Cellular proliferation

The MTS assay was used to determine whether our sigma receptor ligands affected cellular metabolism in MDA-MB-468 cells, which express both sigma-1 and sigma-2 receptors and MCF7 cells, which appear to express only sigma-2 receptors. All but one of the straight-chain ammonium salts were unable to decrease cell proliferation at concentrations 10 times above the K_i_ for the relevant receptor (data not shown). Due to its high affinity, dioctylammonium was tested at concentrations well above its K_i_ and it was found able to reduce cellular proliferation, albeit with pIC_50_ value of 5.0 ± 0.2, well above our reported affinity (pK_i_ for the low affinity state 9.4).

**Figure 1:**
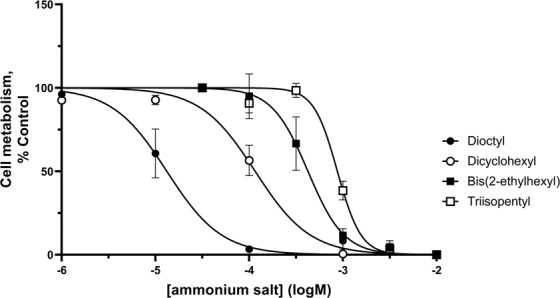
Certain ammonium salt sigma-1 receptor ligands reduce cellular metabolism in MDA-MB-468 cells. Dioctylammonium (n = 4, filled circles), dicyclohexylammonium (n = 4, open circles), bis(2-ethylhexyl)ammonium (n = 6, filled squares) and triisopentylammonium (n = 6, open squares) reduced cellular activity in a concentration-dependent manner. Error bars show SEM. This figure is modified from the PhD thesis by J.M. Brimson^44^.

**Figure 2:**
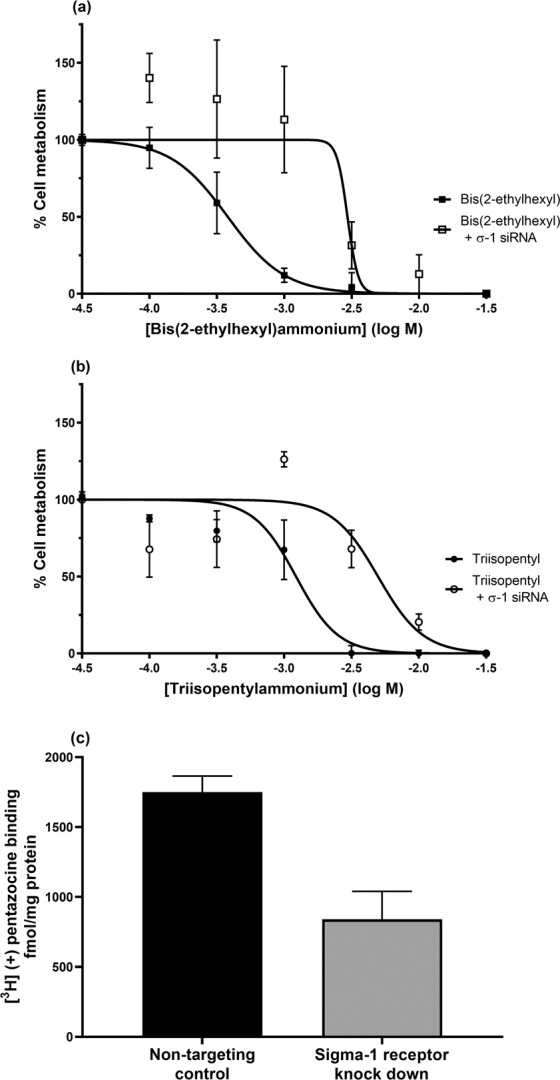
Sigma-1 receptor siRNA hinders the ability of (**a**) bis(2-ethylhexyl)ammonium and (**b**) triisopentylammonium to reduce cellular metabolism in MDA-MB-468 cells. Levels of binding of 30 nM [³H] (+) pentazocine (**c**) are also shown. SiRNA targeting the sigma-1 receptor caused a shift in the pIC_50_ for bis(2-ethylhexyl)ammonium from 3.37 to 2.7 (*P* = 0.044, unpaired t-test), and for triisopentylammonium from 2.9 to 2.3 (*P* = 0.0095, unpaired t-test). Error bars show SEM, n = 3. SiRNA also caused a reduction in the sigma-1 receptor number, as monitored using a fixed concentration (30 nM) of [³H] (+) pentazocine (*P* < 0.0001, unpaired t-test, n = 8).This figure is reproduced from the PhD thesis by J.M. Brimson^44^. Panel (c) has been previously presented^19^.

The branched-chain ammonium salts, bis(2-ethylhexyl)ammonium (pIC_50_ 3.3 ± 0.1), dicyclohexylammonium (pIC_50_ 3.9 ± 0.1) and triisopentylammonium (pIC_50_ 2.96 ± 0.06) caused a dose-dependent reduction in cell metabolism in sigma-1 receptor-expressing MDA-MB-468 cells (Fig. 1). They were without effect on MCF7 cells. This suggests their effects were mediated through the sigma-1 receptor and not the sigma-2 receptor. Furthermore, sigma-1 receptor knock down, using a sigma-1 receptor targetting siRNA, caused a shift in the pIC_50_ values for both bis(2-ethylhexyl)ammonium and triisopentylammonium, from 3.37 ± 0.08 to 2.7 ± 0.2 and 2.9 ± 0.2 to 2.3 ± 0.2 respectively (Fig. 2). Under these conditions, non-targeting control showed 1750 ± 115 fmol.mg^−1^ (mean ± SEM) (+) pentazocine binding; sigma-1 siRNA showed 840 ± 200 fmol.mg^−1^ ± SEM (+) pentazocine binding. The mean difference in binding from 8 independent experiments was 911 ± 151 fmol.mg^−1^. Unpaired t test analysis gave *P* < 0.0001 (Fig. 1c and^19^).

### Calcium response

MDA-MB-468 cells were loaded with fluorescent Fura-2 AM (Supplementary Methods). The branched-chain ammonium salt, bis(2-ethylhexyl)ammonium, did produce a dose-dependent calcium response, with pEC_50_ = 2.9 ± 0.1 (n = 6) (Supplementary Fig. SF7). Triisopentlyammonium also showed a calcium response at concentrations 1 mM and above (data not shown). No other ammonium salt was able to increase cytoplasmic calcium, even at a concentration 100 times the K_i_ (data not shown). We were unable to assess the calcium response to dioctylammonium, as precipitation was observed in the calcium buffer (data not shown).

### Ammonium salt specificity for the sigma receptors

Having determined that these simple ammonium salts are non-toxic *in vitro* and some have high affinity for the sigma receptors, we sought to determine whether these agents also bound other targets. We chose to study dipentylammonium and tripentylammonium salts from the straight-chained ammonium salts and bis(2-ethylhexyl)ammonium and triisopentylammonium from the branched-chain ammonium salts. In terms of “druglikeness”, the free amine dipentylamine fits well into the limitations proposed by Lipinski^21^, including a partition coefficient (logP) value of 3.82. In contrast, tripentylamine, bis(2-ethylhexyl)amine and triisopentylamine, have logP values above 5 (6.44, 6.64 and 5.89, respectively) (ACD prediction, (www.chemspider.com)), outside the Lipinski guidelines. The partition coefficients of the ammonium salts at pH 7.4 (logD (7.4)) was as follows: dipentylammonium 0.65; tripentylammonium 3.73; bis(2-ethylhexyl)ammonium 3.38 and; triisopentylammonium 2.68 (ACD prediction, (www.chemspider.com)). According to Clark’s 5 “rules of thumb” a logD (7.4) of 1–3 is favoured. In addition, he suggests that a charge of +1 aids access to the brain^22^.

### Muscarinic receptors

MDA-MB-468 cells do not express muscarinic receptors. We used rat brain homogenates to assess muscarinic receptor binding. The non-selective muscarinic antagonist, atropine, bound with high affinity (pK_i_ 9.4 ± 0.3, n = 4). Dipentylammonium (pK_i_ 3.4 ± 0.2, n = 5) and tripentylammonium (pK_i_ 4.3 ± 0.4, n = 4) showed low affinity for the muscarinic receptor, similar to rimcazole (pK_i_ 4.5 ± 0.1, n = 7) and 1-(4-iodophenyl)-3-(2-adamantyl)guanidine (IPAG), a second sigma-1 receptor antagonist (pK_i_ 4.3, n = 1). Bis(2-ethylhexyl)ammonium (pK_i_ 3.9 ± 0.2, n = 6) and triisopentylammonium (pK_i_ 3.60 ± 0.11, n = 5) also had similar affinities. Of the ammonium salts tested, the straight-chained ammonium salts dipentylammonium and tripentylammonium were the most selective for the sigma-1 receptor over the muscarinic acetylcholine receptor. Of the branched-chain ammonium salts, triisopentylammonium was more selective than bis(2-ethylhexyl)ammonium (Table 2). Dipentylammonium, tripentylammonium and triisopentylammonium were more selective for sigma-1 receptors than rimcazole (Table 2).

**Table 2:** Selectivity of sigma-1 receptor ligands for the sigma-1 receptor (σ-1 R) over the muscarinic acetylcholine receptor (mACh R).

Compound	σ -1 R affinity pK_i_ ± SEM (n)	σ -1 R affinity K_i_ (µM)	mACh R affinity pK_i_ ± SEM (n)	mACh R affinity K_i_ (µM)	mACh R K_i_/σ-1 R K_i_
Atropine	4.5 ± 0.4 (4)	31	9.4 ± 0.3 (4)	0.0004	0.000012
Rimcazole	6.2 ± 0.2 (7)	0.60	4.49 ± 0.10 (7)	32	53
Dipentyl ammonium	7.4 ± 0.5 (6)	0.043	3.4 ± 0.2 (5)	380	8840
Tripentyl ammonium	7.8 ± 0.2 (6)	0.015	4.3 ± 0.4 (4)	47	3130
Bis(2-ethylhexyl) ammonium	4.5 ± 0.3* (5)	29	3.9 ± 0.2 (6)	120	4.1
Triisopentyl ammonium	4.1 ± 0.4* (6)	79	3.60 ± 0.11 (5)	251	3.4

Affinities for the sigma-1 and muscarinic acetylcholine receptors (σ-1 R and mACh R, respectively) are shown as pK_i_ and K_i_ values from competition binding assays. mACh R K_i_/σ-1 R K_i_ shows the ratio between K_i_ values. These data are reproduced from the PhD thesis by J.M. Brimson^44^.

### Straight-chained ammonium salts as sigma-1 receptor agonists and antidepressants

We have determined that our simple ammonium salts show moderate to high affinity for the sigma receptors. Based on MTS data with MCF7 cells, they appear to show no toxicity. We have, therefore assessed these compounds for their behavioural effects using the forced-swim test^23^ and the tail suspension test^24^, the most used animal models for screening antidepressants.

**Figure 3:**
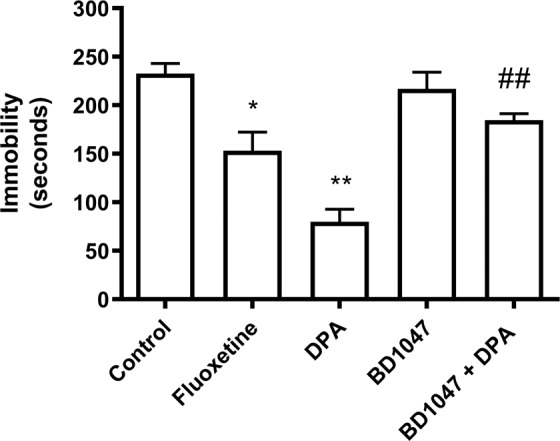
The effect of dipentylammonium (DPA, 5 mg.kg^−1^) and sigma-1 receptor antagonist BD1047 (4 mg.kg^−1^) on despair behaviour in mice subjected to the forced-swim test. The control consisted of 10 ml.kg^−1^ saline; fluoxetine (10 mg.kg^−1^) was used as a positive control. Five mice were used for each data point. Error bars show SEM. ANOVA followed by Bonferroni *post hoc* multiple comparison gave: *control vs fluoxetine, *P* 0.0022; **control vs dipentylammonium, *P* < 0.0001; control vs BD1047, *P* = 0.13; ^##^dipentylammonium vs BD1047 + dipentylammonium, *P* = 0.0001. This figure is reproduced from the PhD thesis by J.M. Brimson^44^.

Five groups of animals (5 animals per group) were submitted to the forced-swim test. Control mice (10 ml.kg^−1^ saline) exhibited despair behaviour, remaining still (only making movements to keep their head above the water) for 233 ± 10 seconds out of a total of six minutes in the water. The selective serotonin reuptake inhibitor, fluoxetine (10 mg.kg^−1^), significantly reduced the time spent immobile to 153 ± 19 seconds (*P* = 0.0022). Similarly, dipentylammonium (5 mg.kg^−1^) significantly reduced the time spent immobile to 80 ± 13 seconds (*P* < 0.0001) (Fig. 3). The sigma-1 receptor antagonist N’-[2-(3,4-dichlorophenyl)ethyl]-N,N,N’-trimethylethane-1,2-diamine (BD1047) alone at 4 mg.kg^−1^ had no significant effect, immobility time being 217 ± 17 seconds (*P* > 0.99). However, a 4 mg.kg^−1^ dose of the sigma-1 receptor antagonist BD1047 prevented a 5 mg.kg^−1^ dose of dipentylammonium from having any effect, immobility time was 185 ± 5 seconds (*P* = 0.0001, compared to dipentylammonium alone), indicating that the effects of dipentylammonium were reversed (Fig. 3). In contrast, tripentylammonium did not have any significant effect on the despair like behaviour of the mice (at doses up to 10 mg.kg^−1^), as measured by the immobility time in the forced-swim test, and was therefore not tested further.

**Figure 4:**
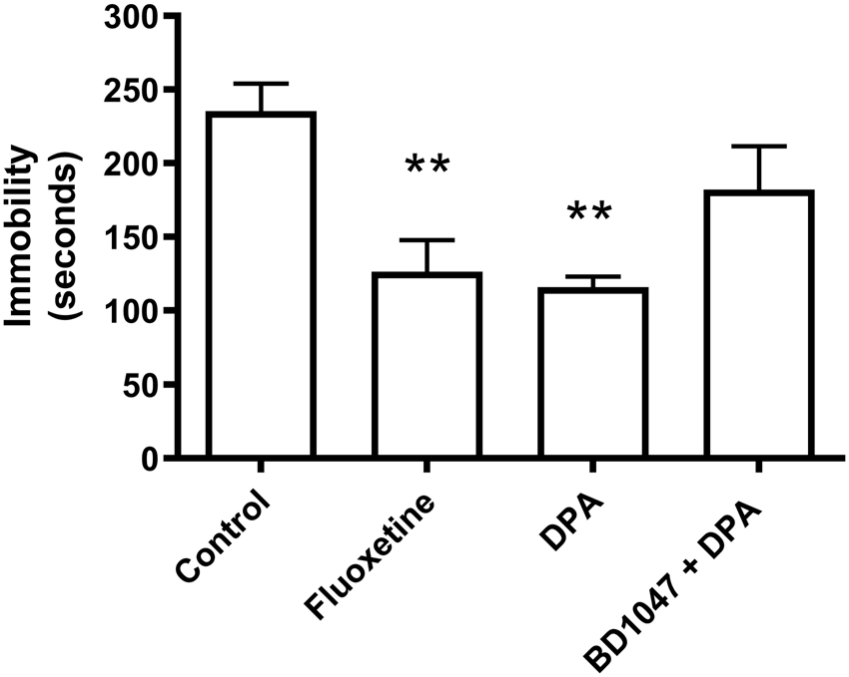
The effect of dipentylammonium (DPA, 5 mg.kg^−1^) and sigma-1 receptor antagonist BD1047 (4 mg.kg^−1^) on despair behaviour in mice subjected to the tail suspension test. The control consisted of an injection of 10 ml.kg^−1^ saline; fluoxetine (10 mg.kg^−1^) was used as a positive control. Ten mice were used for each data point, except BD1047 + dipentylammonium (n = 5). Error bars represent SEM. ANOVA followed by Bonferroni’s *post hoc* multiple comparison gave: **control vs fluoxetine *P* = 0.005, **control vs dipentylammonium, *P* = 0.0001; control vs BD1047 + dipentylammonium, *P* = 0.358; dipentylammonium vs BD1047 + dipentylammonium, *P* = 0.15. This figure is reproduced from the PhD thesis by J.M. Brimson^44^.

Dipentylammonium was also tested in the tail suspension test, in which four groups of mice were suspended by their tails for a period of six minutes. Despair behaviour is interpreted as time spent completely immobile (not struggling to escape). Control mice (10 ml.kg^−1^ saline, n = 10) exhibited despair behaviour remaining immobile for 235 ± 19 seconds (Fig. 4). As with the forced-swim test, treatment with fluoxetine (10 mg.kg^−1^, n = 10) resulted in a significant decrease in time spent immobile (126 ± 22 seconds, *P* = 0.005). Dipentylammonium (5 mg.kg^−1^, n = 10) also resulted in a significant decrease in time (116 ± 7 seconds, *P* = 0.0001). Injecting the sigma-1 receptor antagonist BD1047 (4 mg.kg^−1^) partially reversed the response to dipentylammonium (5 mg.kg^−1^) (n = 5), resulting in an immobility time of 182 ± 30 seconds (*P* = 0.358, compared to control). Dipentylammonium alone (5 mg.kg^−1^) or in combination with BD1047 (4 mg.kg^−1^) had no effect on locomotion (control 140 ± 15 events in 15 minutes, dipentylammonium 128 ± 19 events in 15 minutes (*P* = 0.82), dipentylammonium plus BD1047 114 ± 14 events in 15 minutes (*P* = 0.43), data not shown).

### Effects on monoamine transporters

**Figure 5:**
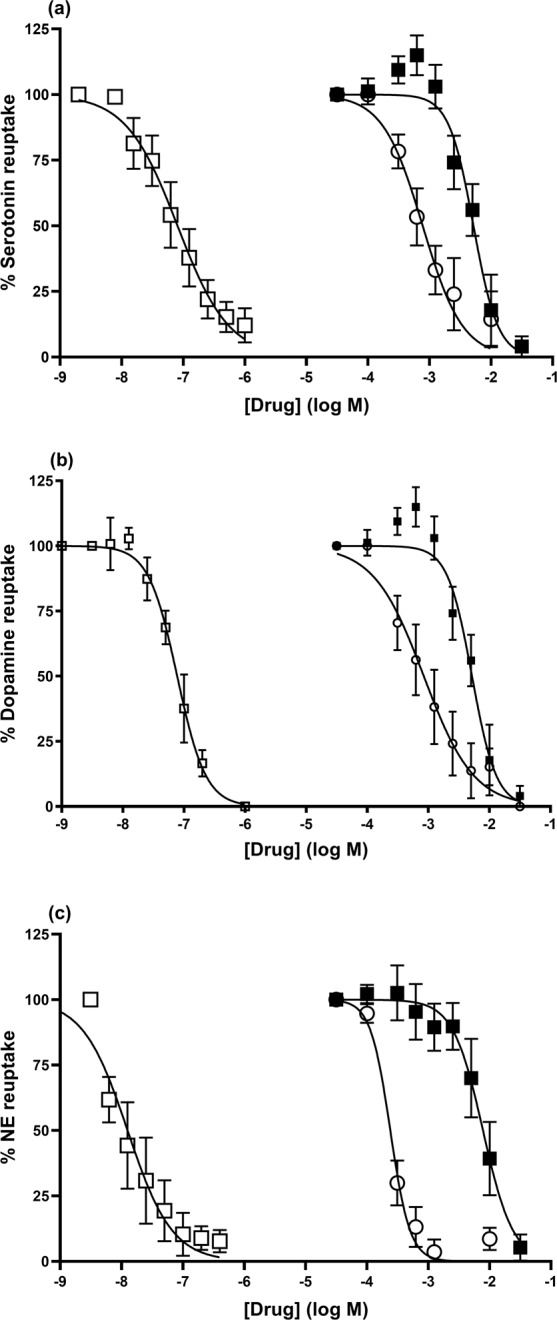
The effects of dipentylammonium and triisopentylammonium on serotonin, dopamine and norepinepherine reuptake. (**a**) fluoxetine (open squares) dose-dependently prevents serotonin reuptake (IC_50_ 83 nM), whereas dipentylammonium (open circles) and triisopentylammonium (closed squares) required a far higher concentration to prevent any serotonin uptake (IC_50_ 1.1 mM and IC_50_ 7.8 mM respectively). (**b**) GBR-12935 (open squares) inhibited dopamine transport with IC_50_ of 76 nM whereas dipentylammonium (open circles) and triisopentylammonium (closed squares) required a far higher concentration to prevent any dopamine uptake (IC_50_ 0.6 mM and IC_50_ 1.6 mM respectively). (**c**) Desipramine (open squares), potently inhibited norepinepherine (NE) reuptake with IC_50_ 9 nM, whereas dipentylammonium (open circles) and triisopentylammonium (closed squares) required a far higher concentration to prevent any norepinepherine uptake (IC_50_ 0.2 mM and IC_50_ 7.8 mM respectively).

The observation that dipentylammonium was effective *in vivo* led us to determine whether it acts on amine transporters. In contrast to fluoxetine, dipentylammonium was found to act very weakly in serotonin reuptake assays. The IC_50_ for fluoxetine (pIC_50_ 7.1 ± 0.1, n = 6, IC_50_ 83 nM) was similar to previously published data (87 nM^25^), whereas dipentylammonium was some 7,000-fold weaker (pIC_50_ 3.0 ± 0.2, n = 4, IC_50_ 1.1 mM) (Fig. 5a). Equally, dipentylammonium was ineffective against the dopamine transporter (pIC_50_ 3.2 ± 0.2, n = 5, IC_50_ 0.6 mM), unlike GBR-12935 which inhibited dopamine transport (pIC_50_ 7.12 ± 0.06, n = 6, IC_50_ 76 nM), in close agreement with previously published results (50 nM^26^) (Fig. 5b). Finally, dipentylammonium was tested against the noradrenaline transporter. In contrast to desipramine, which potently inhibited noradrenaline transport (pIC_50_ 8.05 ± 0.14, n = 3, IC_50_ 9 nM), dipentylammonium interacted weakly (pIC_50_ 3.6 ± 0.1, n = 3, IC_50_ 0.2 mM) (Fig. 5c). The data for desipramine are in close agreement with previously published data (K_i_ 4.3 nM^27^).

### Effects of branched-chain ammonium salts of tumour growth

#### MAC13 assay; *in vitro* assay

**Figure 6:**
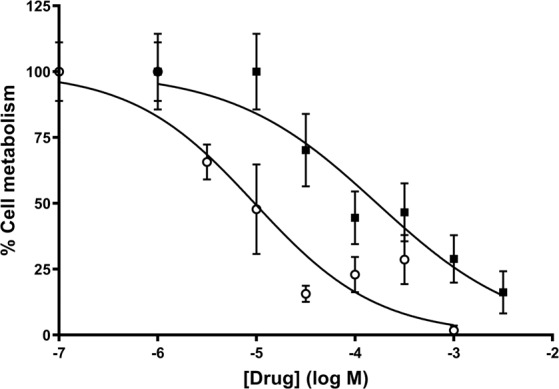
The effects of bis(2-ethylhexyl)ammonium and triisopentylammonium on metabolism of MAC13 cells in the MTS assay. Bis(2-ethylhexyl)ammonium (open circles) had a mean pIC_50_ of 5.3 ± 0.2, and triisopentylammonium (filled squares) had a mean pIC_50_ of 4.4 ± 0.2 from 6 independent assays. This figure is modified from the PhD thesis by J.M. Brimson^44^.

We have shown above that bis(2-ethylhexyl)ammonium and triisopentylammonium both cause a reduction of metabolic activity of MDA-MB-468 cells in the MTS cell survival assay. We subsequently wanted to test their effects on tumours *in vivo* using MAC13 cell transplant^28^. Initially MAC13 cells were tested for sigma 1 receptor expression, using radioligand saturation binding. [^3^H] (+) pentazocine saturation resulted in mean ± SEM B_max_ of 1310 ± 140 fmol.mg^−1^, and the mean ± SEM pK_d_ was 7.4 ± 0.1 (n = 3). Both bis(2-ethylhexyl)ammonium and triisopentylammonium caused a reduction in MAC13 cell metabolism *in vitro* (Fig. 6), with mean ± SEM pIC_50_ (n = 6) values of 5.3 ± 0.2 and 4.4 ± 0.2 respectively. In this respect, MAC13 cells are more sensitive to our ligands than MDA-MB-468 cells (pIC_50_ 3.3 and 2.96, respectively, see above).

#### MAC13 assay; *in vivo* assay

**Figure 7:**
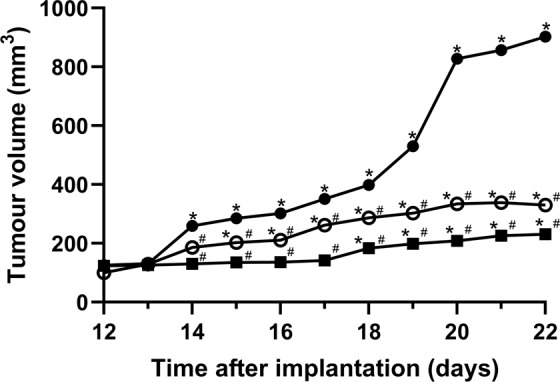
The prevention of MAC13 tumour growth *in vivo* by bis(2-ethylhexyl)ammonium (open circles) and triisopentylammonium (filled squares) compared to vehicle treated mice (filled circles). Drug treatment commenced 12 days after implantation of the tumour. Error bars represent SEM from 6 mice. ^*^Statistically significant tumour growth from day 12, determined using 2-way repeated measures ANOVA with Tukey’s *post hoc* test, *P* < 0.001. ^#^Statistically significant reduction in tumour growth compared to the equivalent day of in the control, *P* < 0.001. This figure is modified from the PhD thesis by J.M. Brimson^44^.

MAC13 tumours were established in the mice 12 days before treatment with the ammonium salts began. Daily treatment with 10 mg.kg^−1^ of bis(2-ethylhexyl)ammonium and triisopentylammonium resulted in a statistically significant reduction in tumour growth compared to vehicle control treated animals (*P* < 0.001) (Fig. 7), suggesting that the two ammonium salts exhibit anti-tumour properties similar to that seen with the sigma-1 receptor antagonists IPAG and rimcazole^11,12^. In addition, the animals showed no negative signs to the use of these drugs: grooming activity was maintained during the study (data not shown).

## Discussion

We have performed structure-activity relationship study using 34 simple ammonium salts for sigma-1 and sigma-2 receptor affinity. Our molecules are far more simple than the pharmacophore model previously proposed for the sigma-1 receptor^29^. The major findings of this study were that several salts show high affinity for these receptors. Selectivity between the ammonium salts ranged from 245-fold preference for the sigma-1 receptor (dioctylammonium) to a 200-fold preference for the sigma-2 receptor (triisopentylammonium). We also identified branched-chained ammonium salts have lower affinities than the straight-chained ammonium salts for the sigma-1 receptor. However, branched-chain ammonium salts, along with the outlier dioctylammonium, showed a binding profile more in line with what has been previously seen with antagonists such as IPAG and rimcazole^19^. The binding profile of all-but-one (dioctylammonium) of the straight-chained ammonium salts showed a simple profile with Hill slope of unity, whereas branched-chain ammonium salts bound with a low Hill slope. This appears to be a novel means to distinguish antagonists from agonists using *in vitro* binding assays and identifies most straight-chained ammonium salts as agonists and the branched-chain ammonium salts as antagonists at this receptor. Further evidence is provided by the observation that, while binding the receptor, the straight-chained ammonium salts did not effect calcium influx or reduce metabolic activity (excepting dioctylammonium, which reduced metabolic activity; we were unable to obtain calcium data for this compound, as precipitation was observed in this assay), whereas some of the branched-chain ammonium salts did, as is the case for antagonists^11^.

Quaternary ammonium salts showed very low affinity for both sigma receptors. However, tetrapropylammonium and tetrabutylammonium are frequently used at mM concentrations for blocking potassium channels^30^ and NMDA channels^31^. The effects of these quaternary ammonium salts on sigma receptors could be relevant when they are being used to block the channels.

It has been shown previously that sigma-1 receptor ligands such as (+) pentazocine and 1 S,2R-cis-N-[2-(3,4-dichlorophenyl)ethyl]-N-methyl-2-(1-pyrrolidinyl)- cyclohexylamine (BD737) are capable of affecting muscarine-induced intracellular calcium changes in SH-SY5Y neuroblastoma cells which have been shown to express the M_1_ and M_3_ subtypes of the muscarinic acetylcholine receptor^32^. These changes could not be reversed by sigma-1 receptor antagonist haloperidol. Therefore, it was deduced that (+) pentazocine (pK_i_ 6.29 ± 0.06^32^) and BD737 were antagonists at the muscarinic receptor. Our binding data suggest that these simple ammonium salts have lower affinity for the muscarinic receptor than rimcazole and (+) pentazocine, suggesting they are more selective than this widely used ligand. Dipentylammonium has a pK_i_ of 7.4 for the sigma-1 receptor^17^, which is significantly higher than that of rimcazole (pK_i_ 6.5). It also has a significantly lower affinity for the muscarinic receptor with a pK_i_ 3.4 compared to rimcazole with a pK_i_ of 4.5. Therefore, dipentylammonium shows greater selectivity than (+) pentazocine, haloperidol and rimcazole.

We have previously shown that IPAG regulates intracellular calcium in a manner regulated by cholera toxin^19^. The concentrations required to effect calcium signalling (pEC_50_ = 3.9, EC_50_ 123 µM) and reduce cellular metabolic activity (pEC_50_ = 4.6, EC_50_ 24 µM) were significantly higher than the equilibrium binding constants (pK_i_ high 12.8, K_i_ high 175 fM and pK_i_ low 6.8, K_i_ low 168 nM)^19^. We describe similar observations here: Bis(2-ethylhexyl)ammonium, dioctylammonium and triisopentylammonium all required concentrations higher than their binding affinities to effect a decrease in cellular metabolism; only dicyclohexylammonium required similar concentrations. This suggests that the equilibrium binding affinities of certain compounds may not represent the binding affinity to the active form of a receptor. The induction of a high-affinity state accompanied by desensitisation following prolonged incubation with ligands has previously been observed with a limited number of other receptors, including the nicotinic acetylcholine receptor^33^, µ-opiate receptor^34^ and GABA receptor^35^. Whilst outside the remit of this publication, we suggest that these observations are further investigated.

There is much evidence to suggest that the sigma-1 receptor is involved in depressive-like behaviours. Sigma-1 knock-out mice show a depressive-like phenotype^36^ and previous studies have identified sigma-1 receptor agonists, such as igmesine, SKF-10,047, and dehydroepiandrosterone sulphate, have antidepressant-like properties in the forced-swim test assay; furthermore, sigma-1 receptor antagonists such as progesterone or BD1047 reverse these effects^37^. It has previously been shown that dipentylammonium has sigma-1 receptor dependant neuroactive properties^17^. Our *in vivo* results suggest that one of these simple ammonium salts, dipentylammonium, is a potential antidepressant acting through the sigma-1 receptor, since the antidepressant-like effects were reversed by BD1047.

Dipentylammonium required very high doses to have any effects on monoamine transporters, excluding them as targets for the effects observed. Neither dipentylammonium *per se* nor in combination with BD1047 had an effect on locomotion suggesting that neither have psychostimulant or psychoinhibitory effects, providing strong evidence that dipentylammonium causes its antidepressant effects through a sigma-1 receptor-mediated mechanism.

Despite having an affinity similar for the sigma-1 receptor to that of dipentylammonium, tripentylammonium failed to reduce despair behaviour in mice subjected to the forced-swim test. This can be explained by the physicochemical properties of the two salts: dipentylammonium met the guidelines proposed by Lipinski^21^, whereas tripentylammonium did not.

Our *in vitro* data for some ligands suggests that they behave as antagonists at the sigma-1 receptor. The binding profiles of bis(2-ethylhexyl)ammonium and triisopentylammonium mimic other sigma-1 receptor antagonists such as IPAG and rimcazole, with shallow Hill slopes and possible multiple binding states^19^, they are able to induce a calcium response, as are other antagonists such as IPAG^11^. Furthermore, treatment of MDA-MB-468 cells with these branched-chain ammonium salts resulted in a reduction of cellular metabolism, which could be reversed by sigma-1 receptor knock-down using siRNA selective for the sigma-1 receptor. Sigma-1 receptor antagonists IPAG and rimcazole have previously been shown to reduce tumour growth *in vivo*^11^. We were able to carry out *in vivo* studies of tumour growth using MAC13 tumours, implanted into NMRI mice. Both bis(2-ethylhexyl)ammonium and triisopentylammonium were able to prevent tumour growth *in vivo* in much the same was as with other studies using sigma-1 receptor antagonists.

## Conclusion

We have identified a non-toxic, high affinity sigma-1 receptor ligand, dipentylammonium, which possesses potent antidepressant activity in animals *in vivo*. Dipentylammonium shows low affinity for classic monoamine transporters yet high affinity for the sigma-1 receptor. Further development of this molecule should be carried out to determine whether this compound can proceed into clinical trials as an antidepressant. We have also identified 2 potential anticancer agents, which act as sigma-1 receptor antagonists: bis(2-ethylhexyl)ammonium and triisopentylammonium. These too, should be further investigated as to whether they may have clinical use.

## Materials and methods

### General materials

Tissue culture media, antibiotics, trypsin and serum were purchased from Invitrogen (Paisley, UK). IPAG (1-(4-iodophenyl)-3-(2-adamantyl)guanidine), purchased from Tocris Bioscience, Bristol, UK, was dissolved in DMSO. [^3^H] (+) pentazocine ((1 *S*,9 *S*,13 *S*)-1,13-dimethyl-10-(3-methylbut-2-en-1-yl)-10-azatricyclotrideca-2,4,6-trien-4-ol) (specific activity 47.6 Ci.mmol^-1^) and [5-^3^H(N)]- 1,3-Di-*O*-tolylguanidine (specific activity 32.2 Ci.mmol^−1^) (DTG) were purchased from Perkin Elmer (Beaconsfield, UK). The simple amines were purchased from Sigma-Aldrich (Dorset, UK), Fluka (Sigma-Aldrich, Dorset, UK) or Acros Organics (Fisher Scientific, Loughborourgh, UK) as either hydrochloride salts (HCl•) or as a free base amines. The free base amines were made soluble in water by making the HCl salt: 5 ml of free base amine was dissolved in 15 ml of ether, and hydrochloric acid was added slowly until the mixture turned acidic, measured using universal indicator paper (Sigma-Aldrich, Dorset, UK). The resulting solid was filtered and any remaining solvent was removed under vacuum. If the product was a liquid at room temperature, the solvents were removed under vacuum. Other reagents were purchased from Sigma-Aldrich (Poole, UK). Before use, drugs were dissolved in an appropriate vehicle and diluted into assay buffer, and the pH of each solution was determined and adjusted to 7.4.

### Tissue culture

MDA-MB-468 and 293 cells (ATCC LGC Promotech, UK) and MCF7 cells (a kind gift from Professor Weiguang Wang, University of Wolverhampton) were maintained in DMEM, high glucose (41965-062) supplemented with 10% foetal calf serum. Cells were cultured at 37 °C in a humidified incubator with 5% CO_2_. The MDA-MB-468 cells were permeabilised (5 pulses at 3.75 KV.cm^−1^) and washed in Tris(hydroxymethyl)aminomethane (Tris) buffered saline (TBS) (10 mM Tris, 0.9% NaCl, pH 7.4) before use in binding assays to remove any endogenous GTP. MCF7 cells were suspended in sigma-2 assay buffer (50 mM Tris-HCl; pH 8.0), sonicated (1 minute) and the homogenate was centrifuged (22,000 *g*, 20 min, 4 °C). The supernatant was discarded, and the pellet was suspended in sigma-2 assay buffer.

### Saturation binding

#### Sigma-1 binding

Assays (100 μl) were performed in TBS using 0–300 nM [^3^H] (+) pentazocine at room temperature for two hours as previously described^17,19^. Non-specific binding was determined using 100 μM rimcazole (9-[3-(*cis*−3, 5-dimethyl-1-piperizinyl)propyl]-9H-carbazole dihydrochloride). Assays were terminated by addition of ice-cold TBS and filtration, using a cell harvester, through glass fibre filters (GF/B, Sigma-Aldrich, Poole, UK). Tubes and filter discs were washed (2 ×3 ml) with ice-cold TBS, and the filter discs dried under vacuum. Scintillation counting was carried out after overnight incubation of the discs with ProSafe FC + cocktail (Meridian Biotechnologies Ltd, Tadworth, UK).

#### Sigma-2 binding

Assays (100 μl) were performed at room temperature for two hours with [^3^H] DTG in the presence of DMSO (10% final concentration). Nonspecific binding was determined using 100 μM reduced haloperidol. No sigma-1 receptor “masking agent” (e.g., (+) pentazocine) was used, as this adversely affects the determination of K_d_ and K_i_ values at the sigma-2 receptor^20^. Assays were terminated by addition of ice-cold sigma-2 wash buffer (150 mM NaCl, 10 mM Tris-HCl; pH 7.2) and filtration, using a cell harvester, through glass fibre filters (GF/C, Sigma-Aldrich, Poole, UK). Tubes and filter discs were washed (2 ×3 ml) with ice-cold wash buffer, and the filter discs dried under vacuum. Scintillation counting was carried out after overnight incubation of the discs with ProSafe FC + cocktail.

### Competition assays

Competition assays (100 μl) were performed using a final assay concentration of 30 nM [^3^H] (+) pentazocine or 10–50 nM [^3^H] DTG. The assay was then allowed to equilibrate at room temperature for four hours. After equilibration, the cells were harvested through GF/B ([^3^H] (+) pentazocine) or GF/C ([^3^H] DTG) glass fibre filters, washing 3 times. Non-specific [^3^H] (+) pentazocine binding was determined using 100 μM rimcazole whereas non-specific [^3^H] DTG binding was determined using 100 μM reduced haloperidol. Under these conditions less than 10% of the [^3^H] (+) pentazocine or [^3^H] DTG was bound.

### Muscarinic receptor binding assays

Rat brain membranes were prepared from freshly killed rats. Rats were exposed to an increasing concentration of carbon dioxide until dead in accordance with Schedule 1 of Animals (Scientific Procedures) Act 1986 and decapitated. This method was approved by Bath University Ethics Committee. The brain was immediately removed and placed on ice with TBS. The brain was homogenised in 10 ml of ice cold TBS by a Teflon-in-glass homogeniser for two 30 s periods, the homogenates were centrifuged at 40,000 *g* for 30 minutes at 4 °C. The resultant supernatant was discarded and the pellets resuspended by homogenisation in fresh TBS. 1.35 nM [^3^H] L-quinuclidinyl [phenyl-4-^3^H] benzilate (specific activity 48.0 Ci.mmol^−1^, GE Healthcare, Little Chalfont, UK) (pK_d_ 9.5 ± 0.1, n = 4 data not shown), was incubated with rat brain membranes (0.5 mg.ml^−1^) to which varying concentrations of ammonium salt compounds were added as with the sigma-1 receptor competition assays. The membranes were incubated and harvested as with the [^3^H] (+) pentazocine binding assays.

### MTS cellular metabolic activity assay

Cells (10,000 cells per well, 100 μl) were seeded into 96-well plates and allowed to adhere overnight. The following day drugs were added and cells were permitted to proliferate for 24 hours before the addition of 10 μl CellTiter 96 AQueous Non-Radioactive Cell Proliferation Assay reagent (Promega, Southampton, UK) as previously described^11^. Absorbance (490 nm) readings were taken at 0, 1, 2 and 3 hours to monitor formazan formation and rates of metabolic activity were compared.

### Vector constructs

The human dopamine transporter gene, kindly provided by Professor De Felice of Virginia Commonwealth University, was released from the pGEX 3Z vector using the *KpnI* and *XbaI* restriction enzymes in a sequential digestion. The DNA fragment was purified by agarose gel electrophoresis, and inserted into the mammalian expression vector, pcDNA3.1, at the *KpnI* and *XbaI* sites. Cloning success was checked using restriction and sequencing analysis.

The human serotonin “Addgene plasmid 15483” and norepinephrine “Addgene plasmid 15475” transporters were obtained in the pcDNA3.1 vector from Addgene^38,39^.

The sigma-1 siRNA PSilencer plasmid and nontargeting siRNA control were kindly provided by Dr Christopher Palmer (London Metropolitan University, UK).

### Transporter studies

Individual 293 cell line clones stably expressing the human dopamine transporter, human 5-HT transporter or human noradrenaline transporter were created using Lipofectamine 2000 (Invitrogen, Gibthai, Bangkok). The 293 cells were plated in 6-well plates at 2.5×10^5^ cells per well, and allowed to adhere overnight. The plasmid constructs were then mixed with Lipofectamine as instructed by the manufacturer and the 293 cells transfected. The following day the cells were harvested, diluted 10-fold and re-plated in 6-well plates before being allowed to adhere overnight. The following day the transfected cells were treated with G418 (Geneticin) (Invitrogen, Gibthai, Bangkok) to begin selection for positive clones. The cells were incubated for 1 to 2 weeks, with the G418-containing media being replaced every 2 days. Once colonies of resistant cells began to appear, they were isolated using sterile cloning rings and transferred to 96-well plates. Each clone was expanded until enough cells were available to verify the expression of the monoamine transporters. The expression was assayed using reverse transcriptase PCR and with the monoamine transporter assay (Molecular Devices, Tokyo Japan).

### Monoamine transporter assay

The 293 cells stably expressing human dopamine, 5-HT or noradrenaline transporters respectively were plated in black-walled 96-well plates at approximately 6,000 cells per well. Once adhered, the media was removed, cells washed with phosphate buffered saline (PBS) and 50 μl Hank’s buffered saline (HBSS) added to each well. Drugs were diluted to 2× concentration in HBSS-0.1% BSA and 50 μl were pipetted into each well. Control wells (cells without any drugs) contained 100 μl HBSS-0.05% BSA. The cells were incubated with the drugs for 30 minutes before the addition of 100 μl per well of fluorescent monoamine analogue dye, which is recognised and transported by all three transporters, and with cell impermeable quencher (homogeneous Neurotransmitter Transporter Uptake Assay Kit, #R8174, Molecular Devices, Tokyo, Japan). The cells were then incubated for a further 15 minutes at 37 °C in the dark before the fluorescence was measured in a microplate reader (excitation: 440 nm, emission: 520 nm). Uptake of the dye was compared to the control wells to calculate the % dye uptake for each drug concentration. Each data point was obtained from at least 3 independent determinations.

### Sigma-1 receptor knock down

Sigma-1 receptor knock down was carried out as previously described^19,40^. Jet-PEI DNA transfection reagent was used to transfect MDA-MB-468 cells, according to the the protocol in the Jet-PEI handbook (Autogen Bioclear, Calne, UK). 24–48 h post transfection sigma-1 receptor activity was assessed. Sigma-1 receptor expression in permeabilised MDA-MB-468 cells was measured using 30 nM [^3^H] (+) pentazocine.

### Test for antidepressant activity

#### Animals

Males from an inbred strain of albino mice (originating from the Laca stock) weighing between 22 and 30 g bred in the Central Animal House (CAH) facility of Panjab University, Chandigarh, were used as previously described^41^. The animals were housed under standard laboratory conditions and maintained on natural light and dark cycle, and had free access to food and water. Animals were acclimatised to laboratory conditions before the experiment. Each animal was used only once. All the experiments were carried out between 09:00 and 15:00 h. The experimental protocols were approved by the Institutional Animal Ethics Committee, Panjab University and conducted according to the Indian National Science Academy Guidelines (INSA) for the use and care of experimental animals.

#### Drugs

All drugs were dissolved in normal saline and different doses were administered intraperitoneally in a fixed volume of 1 ml.100 g^−1^ body weight 30 minutes before the animals were subjected to the test. BD1047 (N’-[2-(3,4-dichlorophenyl)ethyl]-N,N,N’-trimethylethane-1,2-diamine) was used as a sigma-1 receptor antagonist.

#### Forced-swim test

Mice were placed inside a rectangular glass jar (25 × 12 × 25 cm) containing water at a depth of 15 cm maintained at 24 ± 1 °C for a period of 6 minutes as previously described^41^. Each mouse was judged to be immobile when it ceased struggling and remained floating motionless in water, making only those movements necessary to keep its head above water. The total immobility period was recorded and compared to vehicle control group.

#### Tail suspension test

Mice were suspended 58 cm above a table top by using adhesive tape placed approximately 1 cm from the tip of the tail for a period of 6 minutes as previously described^42^. Each mouse was considered immobile when it hung passively and completely motionless. The total immobility period was recorded and compared to vehicle control group.

#### Locomotive activity

Locomotor activity (ambulations) was measured by using a computerised Actophotometer (IMCORP, India). An array of 16 infrared emitter/detector pairs measured animal activity along a single axis of motion, the digital data being displayed on the front panel as ambulatory movements. Mice were allowed to acclimatise to the observation chamber for a period of 2 minutes. The activity was monitored continuously for a period of 15 minutes. Locomotion was expressed in terms of total photobeam counts per 15 minutes per animal and compared to vehicle control groups as previously described^41^.

### *In vivo* cancer mouse model

#### Animals

Naval Medical Research Institute (NMRI) mice weighing 25 g were used for tumour transplantation, they were kept on a 12 hour day-night cycle, at ambient temperature, with free access to food (standard chow diet, Special Diet Services, Lillico, Wonham Mill, Bletchworth Surrey, UK) and water, the consumption of which was monitored throughout the experiments.

#### MAC13 cancer model transplant and treatment

The NMRI mice were transplanted with a fragment of mouse adenocarcinoma (MAC13) in the flank, by means of a trocar^43^. Twelve days after the tumour was implanted, animals (6 animals per group) were treated, with either ammonium salt (10 mg.kg^−1^ i.v., via the tail vein) or control (100 μl PBS i.v., via the tail vein) daily. Animals were terminated before the tumour exceeded 1 cm^³^. The tumour diameters were measured daily from day 12 using a micrometer. Animal studies were conducted under Home Office License according to the U.K. Coordinating Committee on Cancer Research Guidelines for the Care and Use of Laboratory Animals. The experimental protocols were approved by Aston University Ethics Committee.

### Data analysis

Saturation and competition data were analysed using non-linear regression analysis (GraphPad Prism, version 6.0 h for Macintosh) to fit saturation curves and calculate maximal radioligand binding and dissociation constants. In order to give a more true representation of the drugs’ affinities for the receptor, the IC_50_ was converted to a K_i_ (where the Hill slope was near unity) value using the Cheng Prusoff equation. Values were converted into pK_d_ or pK_i_ before any mathematical manipulation to ensure normal distribution. Data are presented as mean ± SEM.

Comparisons of Hill slope were performed using a one-sample t test (GraphPad Prism, version 6.0 h for Macintosh), comparing values to unity. *P* < 0.05 was considered statistically significant.

MTS and protein assays were read using a Versamax plate reader, and the data collected using Softmax Pro software.

Results from behavioural assays and tumour studies are expressed as mean ± SEM. Significance of differences was determined using one-way ANOVA followed by a Dunnett’s (when comparing against a control group) or Tukey’s (when comparing all means) *post hoc* test. *P* < 0.05 was considered statistically significant.

### Protein measurements

Protein amounts were determined using Bio-Rad Protein Dye Reagent, based on Coomassie Brilliant Blue G250 (Bio-Rad, Hemel Hempstead, UK). BSA was used to prepare standards.

## Supplementary information

Supplementary information.

## Data Availability

The datasets generated during and/or analysed during the current study are available from the corresponding author on reasonable request.

## References

[1] Martin WR (1976). The effects of morphine- and nalorphine- like drugs in the nondependent and morphine-dependent chronic spinal dog. J Pharmacol Exp Ther.

[2] Hanner M (1996). Purification, molecular cloning, and expression of the mammalian sigma1-binding site. Proc Natl Acad Sci USA.

[3] Schmidt HR (2016). Crystal structure of the human sigma1 receptor. Nature.

[4] Fontanilla D (2009). The hallucinogen N,N-dimethyltryptamine (DMT) is an endogenous sigma-1 receptor regulator. Science.

[5] Alon A (2017). Identification of the gene that codes for the sigma2 receptor. Proc Natl Acad Sci USA.

[6] Maurice T, Su TP (2009). The pharmacology of sigma-1 receptors. Pharmacol Ther.

[7] Safrany ST, Brimson JM (2016). Are fluoxetine’s effects due to sigma-1 receptor agonism?. Pharmacol Res.

[8] Werling LL, Keller A, Frank JG, Nuwayhid SJ (2007). A comparison of the binding profiles of dextromethorphan, memantine, fluoxetine and amitriptyline: treatment of involuntary emotional expression disorder. Exp Neurol.

[9] Vilner BJ, John CS, Bowen WD (1995). Sigma-1 and sigma-2 receptors are expressed in a wide variety of human and rodent tumor cell lines. Cancer Research.

[10] Caveliers V (2001). Labelled sigma receptor ligands: can their role in neurology and oncology be extended?. Eur J Nucl Med.

[11] Spruce BA (2004). Small molecule antagonists of the sigma-1 receptor cause selective release of the death program in tumor and self-reliant cells and inhibit tumor growth *in vitro* and *in vivo*. Cancer Research.

[12] Wang L (2005). Sigma receptor antagonists inhibit human lens cell growth and induce pigmentation. Invest Ophthalmol Vis Sci.

[13] Baxendale IR, Hayward JJ, Ley SV, Tranmer GK (2007). Pharmaceutical strategy and innovation: An academics perspective. Chemmedchem.

[14] Ablordeppey S, Fischer JB, Glennon RA (2000). Is a nitrogen atom an important pharmacophoric element in sigma ligand binding?. Bioorg Med Chem.

[15] Glennon RA (1994). Structural features important for sigma 1 receptor binding. J Med Chem.

[16] Fujimura K (1997). Synthesis, structure and quantitative structure-activity relationships of sigma receptor ligands, 1-[2-(3,4-dimethoxyphenyl)ethyl]-4-(3-phenylpropyl) piperazines. Bioorg Med Chem.

[17] Brimson JM, Safrany ST, Qassam H, Tencomnao T (2018). Dipentylammonium Binds to the Sigma-1 Receptor and Protects Against Glutamate Toxicity, Attenuates Dopamine Toxicity and Potentiates Neurite Outgrowth in Various Cultured Cell Lines. Neurotox Res.

[18] Ramachandran S (2009). The sigma1 receptor interacts with N-alkyl amines and endogenous sphingolipids. Eur J Pharmacol.

[19] Brimson JM, Brown CA, Safrany ST (2011). Antagonists show GTP-sensitive high-affinity binding to the sigma-1 receptor. Br J Pharmacol.

[20] Abbas, H., et al. Hazards of Using Masking Protocols When Performing Ligand Binding Assays: Lessons From the Sigma-1 and Sigma-2 Receptors. *Frontiers in Pharmacology* **11** (2020). 10.3389/fphar.2020.00309 PMC7082753 32231573

[21] Lipinski CA, Lombardo F, Dominy BW, Feeney PJ (1997). Experimental and computational approaches to estimate solubility and permeability in drug discovery and development settings. Advanced Drug Delivery Reviews.

[22] Clark DE (2003). In silico prediction of blood-brain barrier permeation. Drug Discovery Today.

[23] Porsolt RD, Bertin A, Jalfre M (1977). Behavioral despair in mice: a primary screening test for antidepressants. Arch Int Pharmacodyn Ther.

[24] Steru L, Chermat R, Thierry B, Simon P (1985). The tail suspension test: a new method for screening antidepressants in mice. Psychopharmacology (Berl).

[25] Shank RP (1988). McN-5652: a highly potent inhibitor of serotonin uptake. J Pharmacol Exp Ther.

[26] Chalon S (1999). Pharmacological characterization of (E)-N-(3-iodoprop-2-enyl)-2beta-carbomethoxy-3beta-(4’-methylphenyl)n ortropane as a selective and potent inhibitor of the neuronal dopamine transporter. J Pharmacol Exp Ther.

[27] Bonisch H (1984). The transport of (+)-amphetamine by the neuronal noradrenaline carrier. Naunyn Schmiedebergs Arch Pharmacol.

[28] Bibby MC, Double JA, Mughal MA (1981). Effects of Nandrolone Decanoate on the Toxicity and Anti-Tumor Action of Ccnu and Fu in Murine Tumors. British Journal of Cancer.

[29] Gund TM, Floyd J, Jung D (2004). Molecular modeling of sigma 1 receptor ligands: a model of binding conformational and electrostatic considerations. Journal of Molecular Graphics & Modelling.

[30] Spassova MA, Lu Z (1998). Coupled ion movement underlies rectification in an inward-rectifier K+ channel. Journal of General Physiology.

[31] Sobolevsky AI, Koshelev SG, Khodorov BI (1999). Probing of NMDA channels with fast blockers. Journal of Neuroscience.

[32] Hong W, Werling LL (2002). Binding of sigma receptor ligands and their effects on muscarine-induced Ca^2+^ changes in SH-SY5Y cells. Eur J Pharmacol.

[33] Ochoa EL, Chattopadhyay A, McNamee MG (1989). Desensitization of the nicotinic acetylcholine receptor: molecular mechanisms and effect of modulators. Cell Mol Neurobiol.

[34] Birdsong WT (2013). Increased agonist affinity at the mu-opioid receptor induced by prolonged agonist exposure. J Neurosci.

[35] Chang Y (2002). Desensitization mechanism of GABA receptors revealed by single oocyte binding and receptor function. J Neurosci.

[36] Sabino V (2009). Sigma-1 receptor knockout mice display a depressive-like phenotype. Behav Brain Res.

[37] Urani A (2001). The antidepressant-like effect induced by sigma(1)-receptor agonists and neuroactive steroids in mice submitted to the forced swimming test. J Pharmacol Exp Ther.

[38] Galli A (1995). Sodium-dependent norepinephrine-induced currents in norepinephrine-transporter-transfected HEK-293 cells blocked by cocaine and antidepressants. J Exp Biol.

[39] Ramamoorthy S (1993). Antidepressant- and cocaine-sensitive human serotonin transporter: molecular cloning, expression, and chromosomal localization. Proc Natl Acad Sci USA.

[40] Aydar E, Palmer CP, Klyachko VA, Jackson MB (2002). The sigma receptor as a ligand-regulated auxiliary potassium channel subunit. Neuron.

[41] Dhir A, Kulkarni SK (2007). Involvement of sigma-1 receptor modulation in the antidepressant action of venlafaxine. Neurosci Lett.

[42] Kulkarni SK, Dhir A (2008). On the mechanism of antidepressant-like action of berberine chloride. Eur J Pharmacol.

[43] Bibby MC (1987). Characterization of a transplantable adenocarcinoma of the mouse colon producing cachexia in recipient animals. J Natl Cancer Inst.

[44] Brimson, J. M. University of Bath (2010).

[45] Abbas, H. University of Wolverhampton (2018).

