# Supplementary material for: Simple ammonium salts acting on sigma-1 receptors yield potential treatments for cancer and depression
Source: Sci Rep. 2020 Jun 8;10:9251. doi: 10.1038/s41598-020-65849-6 (PMC7280195; doi:10.1038/s41598-020-65849-6)
Supplement: Supplementary file 1 — Supplementary information. [file 41598_2020_65849_MOESM1_ESM.docx]

Supplementary information.

Simple ammonium salts acting on sigma-1 receptors yield potential treatments for cancer and depression.

James M Brimson^1,2^, Kiran K Akula^3,4^, Haider Abbas^5,6,7^, David R Ferry^8^, Shrinivas K Kulkarni^3^, Steven T Russell^9^ Michael J Tisdale^9^, Tewin Tencomnao^1^ and Stephen T Safrany^2,5,10*^

**Running title:** Ammonium salts, depression and cancer

## Supplementary Methods

## Fura-2 calcium measurements

Approximately 2 x 10^7^ cells were resuspended in 2 ml of buffer comprising: 115mM NaCl, 5mM KCl, 1mM NaH_2_PO_4_, 0.5mM MgSO_4_, 11mM glucose, 1.36mM CaCl_2_, 0.1% BSA, 50mM Tris (note that Tris and not 4-(2-hydroxyethyl)-1-piperazineethanesulfonic acid (HEPES) was used to buffer the cells as HEPES binds the sigma-1 receptor with a K_i_ of 12 mM ^1^) pH 7.4. The cells were loaded with 5 mM Fura-2-acetoxymethyl ester (Fura-2-AM; Invitrogen) for 45 minutes at room temperature in the dark, washed and incubated at room temperature for a further 30 min. The cells were then kept in suspension in the dark at room temperature before use in calcium measurements. Stirred suspensions (2 ml) of Fura-2-loaded cells were placed in a cuvette with a magnetic stirring bar, and maintained at 37 °C. The cells were excited at 340 nm and 380 nm and emission was recorded at 510 nm using a Perkin Elmer LS 50B ﬂuorescence spectrometer. Fura-2 fluorescence data was collected using FL WinLab software (Perkin Elmer, USA). At the end of each individual experiment 20 μl of 1 mg.ml^-1^ digitonin was added to the cuvette to permeabilise the cells allowing calcium to ﬂood the dye giving a maximum ﬂuorescence, followed by 60 μl of 0.5 M EDTA pH 8.5 to chelate the calcium giving a minimum ﬂuorescence. These values were then used to calculate the cytoplasmic calcium concentration of the cells ^2^. Unloaded cells were used to check autoﬂuorescence and drug ﬂuorescence ^1^.

**References**

1. Brimson, J.M., Brown, C.A. Safrany, S.T. Antagonists show GTP-sensitive high-affinity binding to the sigma-1 receptor. *Br J Pharmacol* **164**, 772-80 (2011).

2. Grynkiewicz, G., Poenie, M. Tsien, R.Y. A new generation of Ca^2+^ indicators with greatly improved fluorescence properties. *J Biol Chem* **260**, 3440-50 (1985).

3. Brimson, J.M. University of Bath (2010).

4. Brimson, J.M., Safrany, S.T., Qassam, H. Tencomnao, T. Dipentylammonium Binds to the Sigma-1 Receptor and Protects Against Glutamate Toxicity, Attenuates Dopamine Toxicity and Potentiates Neurite Outgrowth in Various Cultured Cell Lines. *Neurotox Res* **34**, 263-272 (2018).

5. Abbas, H. University of Wolverhampton (2018).

**Supplementary Figure SF1**: Binding of primary ammonium salts to (a) the sigma-1 receptor, as determined using competition binding studies with [³H] (+) pentazocine and permeabilised MDA-MB-468 cells, and (b) the sigma-2 receptor, as determined using competition binding studies with [³H] DTG and membranes prepared from MCF7 cells. Data are presented as % specific binding of radioligand. Error bars represent SEM. Number of independent experiments, pK_i_ and K_i_ values can be found in Supplementary Table ST1.

**Supplementary Figure SF2**: Binding of secondary ammonium salts to (a) the sigma-1 receptor, as determined using competition binding studies with [³H] (+) pentazocine and permeabilised MDA-MB-468 cells, and (b) the sigma-2 receptor, as determined using competition binding studies with [³H] DTG and membranes prepared from MCF7 cells. Data are presented as % specific binding of radioligand. Error bars represent SEM. Number of independent experiments, pK_i_ and K_i_ values can be found in Supplementary Table ST1.

**Supplementary Figure SF3**: Binding of dioctylammonium, bis(2-ethylhexyl) ammonium and triisopentylammonium to the sigma-1 receptor, as determined using competition binding studies with [³H] (+) pentazocine and permeabilised MDA-MB-468 cells. Two-site binding curves show low and high affinity states for the sigma-1 receptor. Data are presented as % specific binding of radioligand. Error bars represent SEM. Number of independent experiments, pK_i_ and K_i_ values, and proportion of each affinity state can be found in Supplementary Table ST1 and the main text.

**Supplementary Figure SF4**: Binding of tertiary ammonium salts to (a) the sigma-1 receptor, as determined using competition binding studies with [³H] (+) pentazocine and permeabilised MDA-MB-468 cells, and (b) the sigma-2 receptor, as determined using competition binding studies with [³H] DTG and membranes prepared from MCF7 cells. Data are presented as % specific binding of radioligand. Error bars represent SEM. Number of independent experiments, pK_i_ and K_i_ values can be found in Supplementary Table ST1.

**Supplementary Figure SF5**: Binding of quaternary ammonium salts to (a) the sigma-1 receptor, as determined using competition binding studies with [³H] (+) pentazocine and permeabilised MDA-MB-468 cells, and (b) the sigma-2 receptor, as determined using competition binding studies with [³H] DTG and membranes prepared from MCF7 cells. Data are presented as % specific binding of radioligand. Error bars represent SEM. Number of independent experiments, pK_i_ and K_i_ values can be found in Supplementary Table ST1.

**Supplementary Figure SF6**: Binding of branched ammonium salts to (a) the sigma-1 receptor, as determined using competition binding studies with [³H] (+) pentazocine and permeabilised MDA-MB-468 cells, and (b) the sigma-2 receptor, as determined using competition binding studies with [³H] DTG and membranes prepared from MCF7 cells. Data are presented as % specific binding of radioligand. Error bars represent SEM. Number of independent experiments, pK_i_ and K_i_ values can be found in Supplementary Table ST1.

**Supplementary Figure SF7.** **Cytoplasmic calcium elevation in response to increasing concentrations of bis(2-ethylhexyl)ammonium**. Bis(2-ethylhexyl)ammonium dose-response curve for a suspension of MDA-MB-468 cells loaded with Fura-2-AM. The pEC_50_ for bis(2-ethylhexyl)ammonium was 2.9 ± 0.1. Error bars show SEM, n = 6. This figure is reproduced from the PhD thesis by J.M. Brimson ^3^.

**Supplementary table ST1. Affinities of the simple ammonium salts tested.**

| Ammonium salt | σ-1 R affinity  pK_i_ ± SEM (n) | σ-1 R affinity  (µM) | σ-2 R affinity  pK_i_ ± SEM (n) | σ-2 R affinity  (µM) | σ-1 R K_i_/  σ-2 R K_i_ | pKa^#^ |
| --- | --- | --- | --- | --- | --- | --- |
| Propyl | <3 (4) | - | <3 (3) | - | - | 10.7 |
| Butyl | <3 (4) | - | 3.26 ± 0.05 (3) | 550 | - | 10.8 |
| Pentyl | <3 (4) |  | 3.9 ± 0.1 (3) | 130 | - | 10.6 |
| Hexyl | 3.5 ± 0.2 (4) | 290 | 5.1 ± 0.2* (3) | 7.8 | 39.8 | 10.2 |
| Octyl | 6.1 ± 0.3 (4) | 0.80 | 5.2 ± 0.2 (3) | 6.2 | 0.13 | 10.2 |
| Decyl | 6.5 ± 0.4 (4) | 0.32 | 5.41 ± 0.07 (3) | 3.9 | 0.081 | 10.6 |
| Dimethyl | <2 (6) | - | <3 (3) | - | - | 10.5 |
| Diethyl | 4.7 ± 0.5 (6) | 19 | <3 (3) | - | - | 10.5 |
| Dipropyl | 5.6 ± 0.2 (6) | 2.6 | 4.10 ± 0.07 (3) | 79 | 32 | 10.7 |
| Dibutyl | 6.9 ± 0.4 (6) | 0.14 | 5.64 ± 0.07 (3) | 2.3 | 18 | 10.7 |
| Dipentyl ^4^ | 7.4 ± 0.5 (6) | 0.043 | 6.9 ± 0.2* (3) | 0.12 | 3.2 | 10.7 |
| Dihexyl | 6.8 ± 0.6 (6) | 0.14 | 7.58 ± 0.06* (3) | 0.026 | 0.17 | 10.7 |
| Dioctyl | 10.3 ± 0.2 * (4) | 0.000050 | 7.9 ± 0.5 (3) | 0.014 | 0.0040 | 10.7 |
| Trimethyl | <2 (6) | - | <3 (3) | - | - | 9.5 |
| Triethyl | 3.4 ± 0.1 (6) | 420 | 3.66 ± 0.04* (3) | 220 | 1.82 | 10.2 |
| Tripropyl | 5.5 ± 0.1 (6) | 3.5 | 4.36 ± 0.07 (3) | 44 | 0.072 | 10.8 |
| Tributyl | 6.9 ± 0.1 (6) | 0.13 | 5.5 ± 0.2* (3) | 3.0 | 0.040 | 10.8 |
| Tripentyl | 7.8 ± 0.2 (6) | 0.015 | 6.1 ± 0.1 (3) | 0.78 | 0.020 | 10.8 |
| Trihexyl | 8.1 ± 0.4 (6) | 0.009 | 6.63 ± 0.03 (3) | 0.23 | 0.034 | 10.8 |
| Trioctyl | 4.7 ± 0.9 (6) | 19 | 3.74 ± 0.06 (3) | 180 | 0.11 | 10.8 |
| Tridodecyl | 4.3 ± 0.1 (6) | 55 | 4.1 ± 0.6 (3) | 76 | 0.63 | 10.8 |
| Tetramethyl | <2 (3) | - | <3 (3) | - | - | - |
| Tetraethyl | 2.3 ± 0.2 (3) | 4,600 | <3 (3) | - | - | - |
| Tetrapropyl | 2.5 ± 0.1 (3) | 3,200 | 3.5 ± 0.2* (3) | 310 | 10 | - |
| Tetrabutyl | 2.8 ± 0.1 (3) | 1,600 | 4.22 ± 0.08* (3) | 60. | 25 | - |
| 1-adamantyl | 3.13 ± 0.06 (6) | 740 | 5.04 ± 0.05* (3) | 9.1 | 83 | 10.7 |
| 2-adamantyl | 3.23 ± 0.09 (6) | 590 | 5.5 ± 0.2 (3) | 3.5 | 190 | 10.5 |
| Diisopropyl | 3.14 ± 0.02 (5) | 720 | 4.1 ± 0.1 (3) | 71 | 16 | 10.7 |
| Diisobutyl | 3.99 ± 0.02 (5) | 100 | 5.2 ± 0.2* (3) | 6.6 | 115 | 11.0 |
| Bis(2-ethylhexyl) | 4.5 ± 0.3* (5) | 29 | 4.0 ± 0.2* (3) | 91 | 0.29 | 11.1 |
| Dicyclohexyl | 5.8 ± 0.7 (5) | 1.7 | 6.86 ± 0.03 (3) | 0.14 | 13 | 11.1 |
| Di-sec-butyl | 3.8 ± 0.3* (3) | 150 | 5.2 ± 0.2 (3) | 6.3 | 24 | 10.9 |
| Triisobutyl | 5.1 ± 0.6 (6) | 7.9 | 5.46 ± 0.06 (3) | 3.5 | 2.3 | 10.8 |
| Triisopentyl | 4.1 ± 0.4* (6) | 79 | 6.40 ± 0.09* (3) | 0.40 | 200 | 10.9 |

Affinities for the sigma-1 and sigma-2 receptors (σ-1 R and σ-2 R, respectively) are shown as pK_i_ and K_i_ values from competition binding assays. Where pK_i_ is shown as <2 or <3, less than 50% of radioligand was competed away by the test solution at 10 mM or 1 mM, respectively. Hill slopes were determined as unity for all salts tested, except those labelled *. σ-1 R K_i_/σ-2 R K_i_ shows the ratio between K_i_ values, where possible. ^#^ Values obtained from chemicalize.com. ^4^ Sigma-1 receptor affinity has been previously published for dipentylammonium. These data are modified from the PhD theses by J.M. Brimson ^3^ and H. Abbas ^5^.
